# Apigenin Isolated from *Carduus crispus* Protects against H_2_O_2_-Induced Oxidative Damage and Spermatogenic Expression Changes in GC-2spd Sperm Cells

**DOI:** 10.3390/molecules27061777

**Published:** 2022-03-08

**Authors:** Spandana Rajendra Kopalli, Sung-Kwang Yoo, Bokyung Kim, Si-Kwan Kim, Sushruta Koppula

**Affiliations:** 1 Department of Bioscience and Biotechnology, Sejong University, Gwangjin-gu, Seoul 05006, Korea; spandanak@sejong.ac.kr; 2 Ottugi Food Co., Ltd., Anyang-si 14060, Gyeonggi-do, Korea; kwangbandco@naver.com; 3 Department of Physiology and Immunology, School of Medicine, Konkuk University, Chungju 27381, Korea; bkkim2@kku.ac.kr; 4 Department of Integrated Biosciences, College of Biomedical & Health Science, Konkuk University, Chungju 27381, Korea; skkim@kku.ac.kr

**Keywords:** welted thistle, apigenin, hydrogen peroxide, reactive oxygen species, testicular cells, nectin, CREB

## Abstract

Testicular oxidative stress is one of the most common factors underlying male infertility. Welted thistle, *Carduus crispus* Linn., and its bioactive principles are attracting scientific interest in treating male reproductive dysfunctions. Here, the protective effects of apigenin isolated from *C. crispus* against oxidative damage induced by hydrogen peroxide (H_2_O_2_) and dysregulation in spermatogenesis associated parameters in testicular sperm cells was investigated. Cell viabilities, ROS scavenging effects, and spermatogenic associated molecular expressions were measured by MTT, DCF-DA, Western blotting and real-time RT-PCR, respectively. A single peak with 100% purity of apigenin was obtained in HPLC conditions. Apigenin treated alone (2.5, 5, 10 and 20 µM) did not exhibit cytotoxicity, but inhibited the H_2_O_2_-induced cellular damage and elevated ROS levels significantly (*p* < 0.05 at 5, 10 and 20 µM) and dose-dependently. Further, H_2_O_2_-induced down-regulation of antioxidant (glutathione *S*-transferases m5, glutathione peroxidase 4, and peroxiredoxin 3) and spermatogenesis-associated (nectin-2 and phosphorylated-cAMP response element-binding protein) molecular expression in GC-2spd cells were attenuated by apigenin at both protein and mRNA levels (*p* < 0.05). In conclusion, our study showed that apigenin isolated from *C. crispus* might be an effective agent that can protect ROS-induced testicular dysfunctions.

## 1. Introduction

Accumulated data from modern medical diagnostics has revealed that both male and female sexes contribute equally to infertility [1]. Moreover, the rate of male subfertility and/or infertility is increasing significantly when compared to that of females [2]. Chemical, environmental, psychological, and physical factors are involved in the occurrence of male sexual disorders including erectile and testicular dysfunctions [3]. It is well understood that oxidative damage induced by uncontrollable generation of reactive oxygen species (ROS) is implicated in the development of sexual and testicular insufficiency [4]. Elevated ROS damages DNA, RNA, and intracellular organelles involved in spermatogenesis [5]. Cumulative evidences indicate that various antioxidants prevent or decrease the pathological changes in testis and moreover inhibit the development of spermacrasia and asthenospermia [6,7]. Further, bioactive molecules from plant origin with immense antioxidant potential have been recognized as promising therapeutic approaches for sexual dysfunction because many chemical drugs are accompanied by a variety of adverse effects [6,7]. In this respect, exploring bioactive molecules originated from plant origin that protect testicular cells from oxidative damage and associated spermatogenesis gene parameters is of great value in the treatment of testicular insufficiency. Further, alternative availability in various plants/natural products of existing compounds of commercial importance is always beneficial, as it will not only ensure future availability, but also value added compounds with better properties can be achieved with high percentage.

Welted thistle, scientifically known as *Carduus crispus* Linn., from the family Asteraceae has long been used as a traditional folk medicine in Korea and other Asian countries to treat various disorders of the liver, bile ducts gallbladder and arthritis [8]. Further, *C. crispus* is known to possess strong anti-oxidative effects [9]. Apigenin, one of the active constituents from milk thistles, was well documented to possess various biological activities including anti-oxidant, anti-inflammatory, neuroprotective and anti-cancer effects [10,11]. 

In relation to male reproductive functions, apigenin exhibited beneficial effects in improving testicular functions in a variety of in vitro and in vivo insults such as heat-, chloroquine-, doxorubicin- and ischemia reperfusion-induced models [4,12,13,14,15,16,17,18,19,20]. However, the effects of apigenin against ROS-induced oxidative damage in sperm cells was not elucidated. Therefore, in the present study, the protective effects of apigenin isolated from *C. crispus* against ROS-induced damage in testicular sperm cells and the regulatory mechanisms involving the spermatogenic gene expression and anti-oxidative enzyme status was investigated using hydrogen peroxide (H_2_O_2_)-induced oxidative damage in GC-2spd sperm cells. 

## 2. Results

### 2.1. Isolation of Apigenin from C. crispus

**Figure 1:**
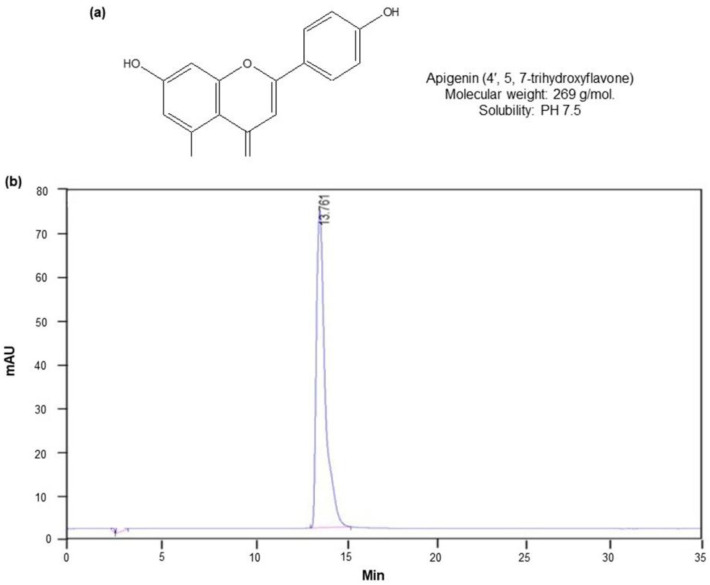
HPLC fingerprint of apigenin isolated from *C. crispus*. (**a**) Chemical name and structure of apigenin. (**b**) HPLC fingerprint of apigenin.

To obtain the active compound apigenin from *C. crispus*, we performed bioassay-guided isolation and purification processes involving the following steps: 100% methanol extraction, EtOAc partition, silica gel column chromatography, preparative HPLC, and re-crystallization in absolute methanol (Figure S1a). The isolated active compound apigenin was proved to be a single peak in optimized HPLC conditions (ODS, 4.6 × 250 mm, 267 nm, 1.0 mL/min, 70% MeOH). A total of 1.5 gm of apigenin was obtained from 10 kg of dried flower bud of *C. crispus* as starting material. Mean peak retention time of apigenin reference material was 14.22 ± 0.097, and content accounted for 100%. No peak derived from contaminants was detected on the HPLC fingerprint with the exception of solvent vehicle at 5 min. The purity of apigenin isolated was found to be 100% when determined by HPLC. The chemical structure and HPLC fingerprint was shown in Figure 1a,b. The purity determination of apigenin (molecular weight: 269) by means of NMR, FT-IR, MS, UV spectral analysis and thin layer chromatography (TLC) was shown in Supplementary Data (Figures S1–S6) and the corresponding data including elemental analysis, optical rotation, linearity and precision analysis data was shown (Tables S2–S6). 

### 2.2. Effect of Apigenin on the Cell Viability in H_2_O_2_-Induced GC-2spd Cells

**Figure 2:**
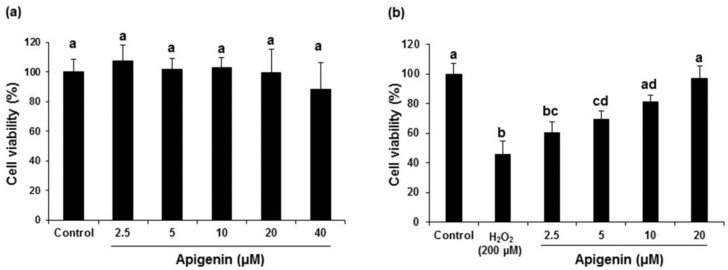
Effects of apigenin on cell viability in GC-2spd sperm cells. Cell viability was evaluated using the MTT assay. (**a**) The effect of apigenin (2.5, 5, 10, 20 and 40 μM) on the viability of GC-2spd cells. (**b**) The effect of apigenin (2.5, 5, 10 and 20 μM) in GC-2spd cells exposed to 200 μM hydrogen peroxide. Data are expressed as the mean ± standard deviation. The groups of control, H_2_O_2_, and different concentrations of apigenin were compared with each other, and letters on the top of the columns that do not share the same letters are statistically significant among the groups (*p* < 0.05) by one-way ANOVA.

Initially, various concentration of apigenin (2.5, 5, 10, 20, and 40 μM) were added to GC-2spd cells and the cell viability was analyzed using MTT assay. Results revealed that apigenin up to 20 μM concentration did not influence the over cell viability in GC-2spd cells. Although, a not significant 40 μM concentration exhibited a mild decrease in viability of GC-2spd cells (Figure 2a). Therefore, the safe and non-toxic concentrations in the range of 2.5, 5, 10, and 20 were selected for further experiments. H_2_O_2_-induced a significant (*p* < 0.05) cytotoxicity in GC-2spd cells (45.60%) when compared with untreated control cells. However, treatment with apigenin inhibited the H_2_O_2_-induced decrease in cell viability significantly (*p* < 0.05 at 5, 10 and 20 μM) in a dose dependent fashion (60.28, 69.34, 81.24 and 96.28% at 2.5, 5, 10, and 20 μM), respectively. Apigenin rescued the H_2_O_2_-induced decrease in cell viability to near normal levels at 20 μM concentrations (Figure 2b). 

### 2.3. Effect of Apigenin on the Intracellular ROS Generation in H_2_O_2_-Induced GC-2spd Cells

**Figure 3:**
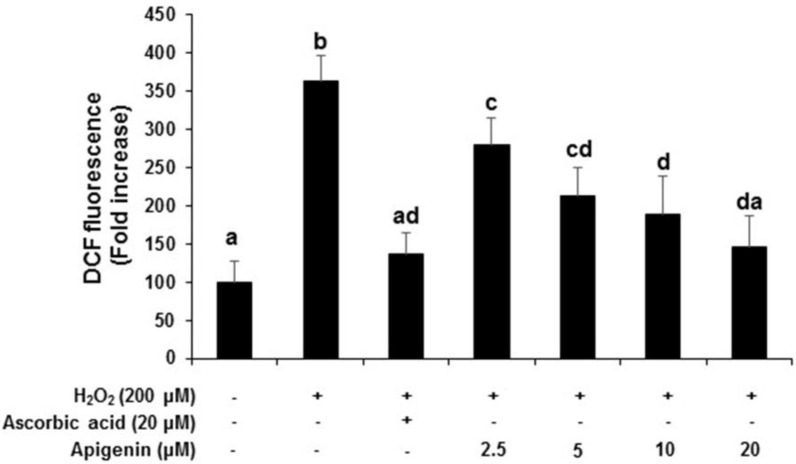
Effect of apigenin on the inhibition of intracellular ROS formation. DCFH-DA fluorescence assay was performed and the fluorescence intensity (fold increase) in each treated group compared with control was shown. H_2_O_2_ (200 μM) treatment significantly increased the fluorescent intensity (fold increase) compared to control cells. Apigenin (2.5, 5, 10 and 20 μM) and positive control ascorbic acid (20 μM) treatment inhibited the H_2_O_2_-exposed increase in ROS generation by decreasing the florescence intensity in GC-2spd cells. Data are expressed as the mean ± standard deviation. The groups of control, H_2_O_2_, ascorbic acid, and different concentrations of apigenin were compared with each other and letters on the top of the columns that do not share the same letters are statistically significant among the groups (*p* < 0.05) by one-way ANOVA.

Intracellular ROS scavenging activity of apigenin was determined using DCF-DA fluorescence assay. H_2_O_2_ exposed to GC-2spd cells exhibited a significant (*p* < 0.05) increase in ROS levels which corresponds to an increase in the fluorescence intensity when compared with untreated control cells (363.63-fold increase). However, the generation of ROS under H_2_O_2_ exposure was significantly (*p* < 0.05) and dose-dependently decreased when treated with apigenin at indicated concentrations (279.90, 213.63, 188.64, 145.46-fold decrease at 2.5, 5, 10, and 20 μM), respectively. The positive control ascorbic acid (20 μM) also significantly (*p* < 0.05) inhibited the H_2_O_2_-induced increase in ROS (136.34-fold decrease) and was comparable to apigenin treated at 20 μM concentration (Figure 3). 

### 2.4. Effect of Apigenin on the Expression of Antioxidant Enzymes in H_2_O_2_-Induced GC-2spd Cells

**Figure 4:**
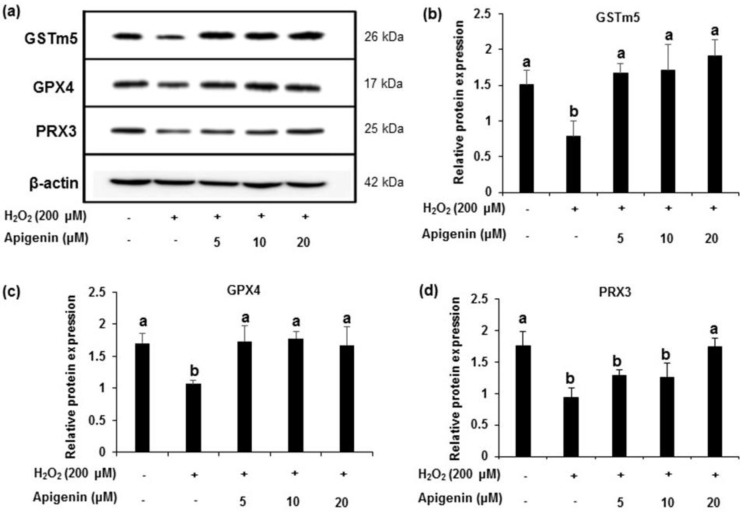
The effect of apigenin on the antioxidant enzyme protein expression level in H_2_O_2_-exposed cells. (**a**) The protein expression of GSTm5, GPX4 and PRX3 in H_2_O_2_-exposed GC-2spd cells was analyzed using Western blotting. Cell lysates from each groups were immunoblotted with specific antibodies. (**b**–**d**) The protein band intensity of GSTm5, GPX4 and PRX3 in H_2_O_2_-exposed GC-2spd cells, respectively, normalized to that of β-actin is shown. The data represent the mean ± standard deviation. The groups of control, H_2_O_2_, and different concentrations of apigenin were compared with each other and letters on the top of the columns that do not share the same letters are statistically significant among the groups (*p* < 0.05) by one-way ANOVA. GSTm5, glutathione S-transferase m5; GPX4, glutathione peroxidase 4; PRX3, peroxiredoxin 3.

**Figure 5:**
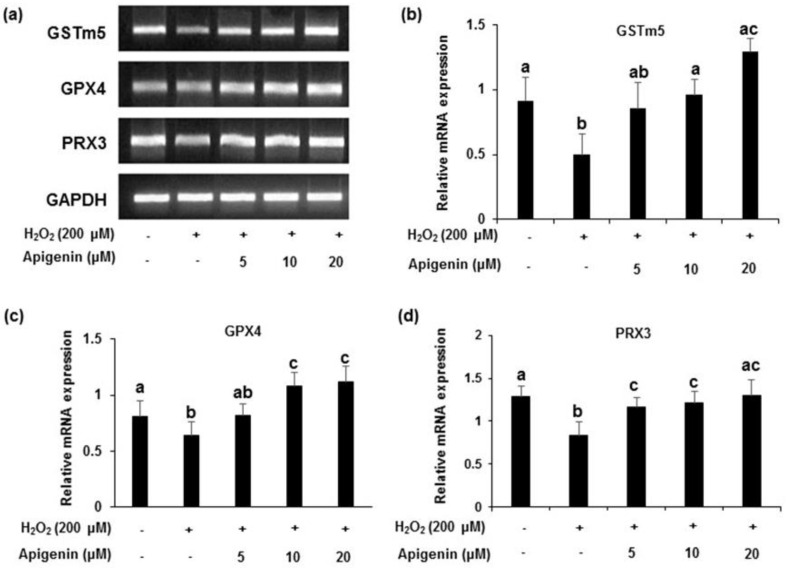
Effects of apigenin on the antioxidant enzyme mRNA expression level in hydrogen peroxide-exposed GC-2spd cells. (**a**) The mRNA expression level of the antioxidant enzymes GSTm5, GPX4 and PRX3 in H_2_O_2_-exposed GC-2spd cells. (**b**–**d**) The polymerase chain reaction band intensity of GSTm5, GPX4 and PRX3 was analyzed using the ImageJ 1.41o software package and was normalized to that of glyceraldehyde 3-phosphate dehydrogenase. Data are expressed as the mean ± standard deviation. The groups of control, H_2_O_2_, and different concentrations of apigenin were compared with each other and letters on the top of the columns that do not share the same letters are statistically significant among the groups (*p* < 0.05) by one-way ANOVA. GSTm5, glutathione S-transferase m5; GPX4, glutathione peroxidase 4; PRX3, peroxiredoxin 3; GAPDH, glyceraldehyde 3-phosphate dehydrogenase.

The protein levels of GSTm5, GPX4 and PRX3 showed a down regulation of expression in H_2_O_2_-induced GC-2spd cell (Figure 4a). All of the enzymes markedly recovered by the treatment with apigenin at indicated concentrations (5, 10, and 20 μM). Band intensities revealed that the relative protein expression of the enzymes significantly (*p* < 0.05) reduced in H_2_O_2_ exposed GC-2spd cells. However, treatment with apigenin attenuated the decreased expression caused by H_2_O_2_ in GC-2spd cells (*p* < 0.05 at 5, 10 and 20 μM for GSTm5 and GPX4; *p* < 0.05 at 20 μM for PRX3) which was significant (Figure 4c). A similar pattern was observed in the mRNA expression of GSTm5, GPX4 and PRX3 in H_2_O_2_-induced GC-2spd cells. Band intensities revealed that apigenin at 10 and 20 μM concentrations exhibited significant effects (*p* < 0.05) and the effects were near normal to the control group (Figure 5a–d). 

### 2.5. Effect of Apigenin on the Expression of Nectin-2 in H_2_O_2-_Exposed GC-2spd Cells

**Figure 6:**
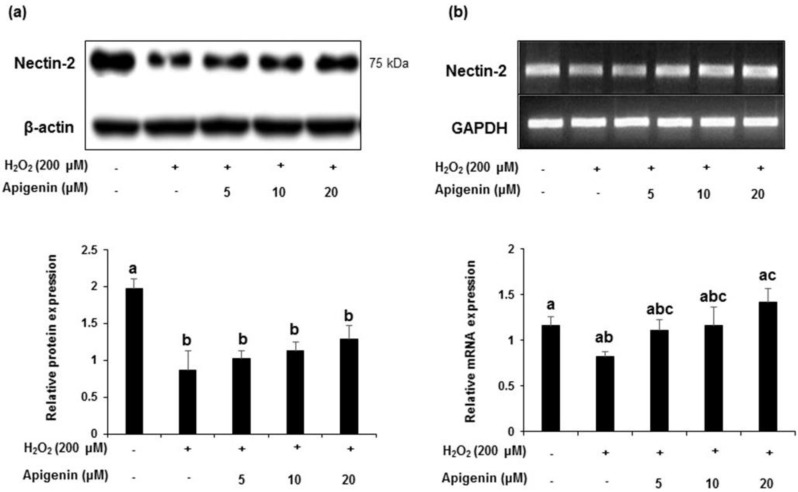
The effect of apigenin on the expression level of nectin-2 in H_2_O_2_-exposed GC-2spd cells. (**a**) The protein expression level of nectin-2 was analyzed using Western blotting. Cell lysates were immunoblotted with specific antibodies with Beta-actin as the internal control (upper panel) and the band intensity of nectin-2 normalized to β-actin is shown in corresponding lower panel. (**b**) The mRNA expression of nectin-2 (upper panel) and corresponding band intensities normalized to GAPDH (lower panel). Data are expressed as the mean ± standard deviation. The groups of control, H_2_O_2_, and different concentrations of apigenin were compared with each other and letters on the top of the columns that do not share the same letters are statistically significant among the groups (*p* < 0.05) by one-way ANOVA.

A decrease in the protein levels of nectin-2 expression was observed when exposed with H_2_O_2_ in GC-2spd cells. However, apigenin treatment ameliorated the decreased expression level of nectin-2 (Figure 6a). Band intensities revealed a significant decrease (*p* < 0.05) in nectin-2 protein expression exposed with H_2_O_2_. Apigenin at indicated concentrations (5, 10 and 20 μM) dose dependently ameliorated these changes (lower lane). With respect to the mRNA levels, a similar pattern was observed and treatment with apigenin (5, 10 and 20 μM) ameliorated these changes dose dependently. Band intensity revealed that apigenin at 20 μM concentration exhibited significant effect (*p* < 0.05) in ameliorating the mRNA expression levels in H_2_O_2_ exposed GC-2spd cells (Figure 6b).

### 2.6. Effect of Apigenin on Expression of CREB in H_2_O_2_ Exposed GC-2spd Cells

**Figure 7:**
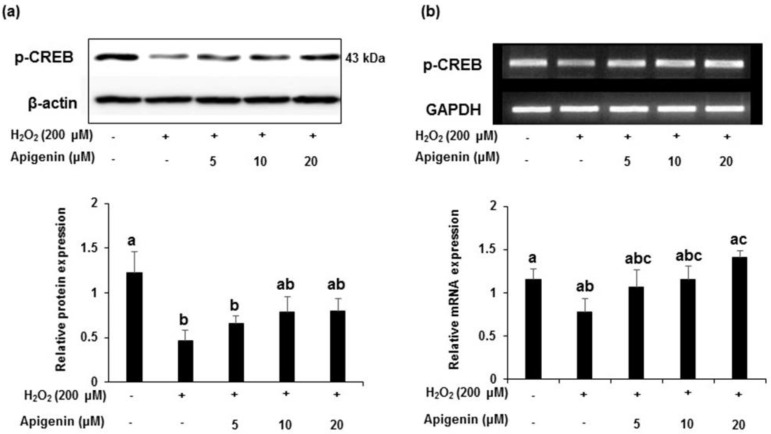
The effect of apigenin on the expression level of p-CREB in H_2_O_2_-exposed GC-2spd cells. (**a**) The protein expression level of p-CREB was analyzed using Western blotting. Cell lysates were immunoblotted with specific antibodies with Beta-actin as the internal control (upper panel) and the band intensity of p-CREB normalized to β-actin is shown in corresponding lower panel. (**b**) The mRNA expression of p-CREB was shown in upper panel and corresponding band intensities normalized to GAPDH was shown in lower panel. Data are expressed as the mean ± standard deviation (*n* = 6). The groups of control, H_2_O_2_, and different concentrations of apigenin were compared with each other and letters on the top of the columns that do not share the same letters are statistically significant among the groups (*p* < 0.05) by one-way ANOVA.

The phosphorylated-CREB protein expression was markedly down-regulated in H_2_O_2_ exposed GC-2spd cells and apigenin at indicated concentrations (5, 10 and 20 μM) recovered these changes (Figure 7a). Band intensities indicated a significant (*p* < 0.05) decrease in the protein expression of *p*-CREB exposed with H_2_O_2_ and apigenin at indicated concentrations (5, 10 and 20 μM) attenuated this decrease dose dependently. Although not significant, a similar pattern was observed in the expression of mRNA levels treated with apigenin in H_2_O_2_ exposed GC-2spd cells (Figure 7b). Band intensity revealed that apigenin treated at highest concentration (20 μM) exhibited significant effect (*p* < 0.05) in ameliorating the mRNA expression levels in H_2_O_2_ exposed GC-2spd cells. 

## 3. Discussion

Thistles have been a topic of increasing interest regarding their clinical applications and they have been used as a treatment for disorders of the liver and kidney [9,21,22,23]. In the present study, we isolated apigenin from Welted thistle, *C. crispus* in its highest purity following EtOAc partition, silica gel column chromatograph, preferential precipitation, and preparative HPLC fractionation. The purity of the apigenin isolation was found to be 100% with a single peak when determined by HPLC method. In this study, apigenin potently exhibited ameliorating effects on ROS-induced oxidative damage and gene down-regulation associated with testicular function, especially antioxidation and spermatogenesis factors.

Oxidative stress has been known to be an important detrimental factor that affects the development of male fertility [4]. ROS are necessary to maintain normal cell function, but an increasing amount of ROS beyond manageable range can play a malignant role in the function and survival of various cells including sperm cells [24,25]. In the present study, we isolated apigenin via bioassay-guided isolation and purification procedure from *C. crispus* and investigated the beneficial effects against oxidative damage induced by H_2_O_2_ in GC-2spd sperm cells. We found that apigenin inhibited the generation of ROS without cytotoxicity in the concentration up to 20 μM, showing a similar activity level to that of a potent antioxidant, ascorbic acid. It has been reported that oxidative stress is implicated in spermatogenesis and testis diseases occurring in a variety of species [26]. Our results imply that apigenin might play a protective role against diverse oxidative stress-associated diseases in the male reproduction system. 

To explain the cellular mechanism of the beneficial effect of apigenin on H_2_O_2_-induced spermatocyte cellular damage, the mRNA and protein levels of key molecules associated with spermatogenesis that are known to be reduced by oxidative stress were investigated [27,28,29]. Among the major antioxidant enzymes, GSTs are known as a large family of abundant enzymes in a variety of cells. GSTs act as detoxification enzymes that catalyze the conjugation of a wide range of electrophilic compounds to glutathione, a tripeptide found in all mammalian cells [30,31,32]. GSTm5 is selectively expressed in testis and is involved in the pathophysiology of testicular functions [32,33]. Moreover, GPX4 reduces complex lipid hydroxides, even if they are incorporated in biomembranes or lipoproteins [34,35]. In addition to its anti-oxidative activity, GPX4 has been implicated as a structural protein in sperm maturation [36,37]. PRX3 is ubiquitously distributed in the mitochondrial matrix and is an antioxidant enzyme that prevents cellular damages caused by oxidative stress [38,39]. Previously, we reported that PRX3 was down-regulated in aged rats [27]. In this study, we found that apigenin influence the mRNA and protein levels of GSTm5, GPX4, and PRX3 in spermatocytes suppressed by H_2_O_2_. These results suggest that apigenin might help sperm cells recover from oxidative damage through the regulation of protein and mRNA expression related to cellular redox systems, and it may prevent sexual dysfunction. 

Previous studies have shown that steroidogenic acute regulatory protein (StAR) is regulated at the transcriptional level via a cAMP-dependent protein kinase (PKA) signaling cascade within the gonads [40,41,42]. Transactivation of target genes by the cAMP-PKA pathway involves the binding of the cAMP response element within the gene promoter [43,44]. Within the classical cAMP-PKA biochemical pathway, activated PKA can phosphorylate CREB on Ser133, which increases in association with the CREB-binding protein (CBP) coactivator, resulting in epigenetic modification and increased transcription [44,45]. Moreover, nectin-2 is a major protein molecule of Sertoli-germ cell adherents in the seminiferous epithelium and regulates spermatogenesis [46,47]. In the present study, we found that apigenin recovered the diminution of phosphorylated-CREB and nectin-2 in spermatocytes at both protein and mRNA levels potently suppressed by H_2_O_2_. Moreover, there is accumulating evidence that CREB and nectin proteins are intrinsically connected with ROS and antioxidant proteins [48,49,50]. These results indicate that apigenin may contribute to spermatogenesis via its effect on testicular epigenetics. However, further research on in vivo experimental models is needed to clarify the intrinsic and in-depth cellular mechanisms by which apigenin improves testicular dysfunction caused by oxidative stress.

## 4. Materials and Methods

### 4.1. Chemicals and Medium for Cell Culture

Organic solvents were purchased from J.T. Baker (Ulsan, Korea). Dulbecco’s modified Eagle’s medium (DMEM), DMEM with F-12 Ham 1:1 mixture (DMEM/F-12), fetal bovine serum (FBS), and horse serum were procured from Hyclone (South Logan, UT, USA). Hydrogen peroxide (H_2_O_2_), ascorbic acid, dimethyl sulfoxide (DMSO), dichlorofluorescein acetate (DCF-DA) and 3-(4,5-dimethylthiazol-2-yl)-2,5-diphenyltetrazolium bromide (MTT), were purchased from Sigma-Aldrich (St. Luis, MO, USA). RNA-Bee reagent and GoTaq^®®^ Green Master Mix were purchased from AMS Bio (Abingdon, UK) and Promega (Madison, WI, USA), respectively. All the primary antibodies that recognized glutathione *S*-transferase (GST) m5, glutathione peroxidase (GPX) 4, peroxiredoxin (PRX) 3, phosphorylated cAMP response element-binding protein (P-CREB), nectin-2 and β-actin were purchased from Santa Cruz Biotech (Santa Cruz, CA, USA). 

### 4.2. Isolation and Purification of Apigenin from C. crispus

The flower of *C. crispus* was collected in the wild field, South Korea, authenticated by taxonomist at Konkuk University. The shade-dried material (10 kg) was extracted with 8 L of 80% methanol (MeOH) at 70 °C for 4 h with reflux system three times. The MeOH extract was evaporated under reduced pressure and partitioned between EtOAc and the aqueous phase three times. The ethyl alcohol (EtOAc) fractions were pooled, dried in vacuo, and subjected to silica gel flash chromatography (Sigma-Aldrich, St. Louis, MO, USA) with solvent systems of chloroform (CHCl_3_):EtOAc at ratios of 4:1 and 2:1. The fraction containing the active ingredient was recrystallized in absolute MeOH to obtain greenish amorphous powder. The active ingredient was further purified with high performance liquid chromatography (HPLC; ODS, YMC-Pack, 20 × 250 mm, 269 nm, 70% MeOH). 

### 4.3. Cell Culture and Viability Assay

Mouse spermatocyte GC-2spd cell lines were obtained from American type culture collection (Manassas, VA, USA). GC-2spd cells were maintained in DMEM supplemented with 10% FBS. The media contained 1.2 g/L sodium bicarbonate, 15 mM HEPES, 100 IU/mL penicillin, and 100 μg/mL streptomycin. For cell viability, MTT [3-(4,5-dimethylthiazol-2-yl)-2,5-diphenyltetrazolium bromide] assay, was used. GC-2spd cells were seeded at a density of 1.0 × 10^4^ cells/well on 96-well plate containing appropriate media overnight. Apigenin at various concentrations (2.5, 5, 10, and 20 μM) was treated for 24 h and compared with the values of unexposed samples in the control group. After the addition of 10 μL MTT solution (5 mg/mL), the cell were subsequently incubated at 3 °C for 4 h. The formazan crystals formed in the viable cells were dissolved in 100 μL DMSO after the media of the wells were removed. The experiment was performed in triplicate and repeated twice independently. The optical density was read at 570 nm using a microplate reader (Tecan, Männedorf, Switzerland).

### 4.4. Intracellular Reactive Oxygen Species (ROS) Scavenging Assay

Intracellular ROS scavenging activity was determined by DCF-DA assay. GC-2spd cell were seeded on 96-well plate at 1x10^4^ cells/well. Twenty-four hours after plating, the cells were treated with varying concentrations of apigenin and two hours later, 200 μM of H_2_O_2_ was added to the plate for 1 h. After 1 h, 20 μM of DCF-DA solution was added for 45 min and then the fluorescence of 2′7′-dichlorofluorescein was detected at 485 nm excitation and at 535 nm emission using the spectrofluorometer (JEOL, Tokyo, Japan). The experiment was performed in triplicate.

### 4.5. Reverse Transcription-Polymerase Chain Reaction (RT-PCR)

GC-2spd cells were plated at 2 × 10^5^ cells/well and incubated for 24 h. After incubation period, the cells were cultured in the presence of apigenin at various concentration for 2 h, and then stimulated by H_2_O_2_ for 1 h. Total RNA was extracted from the cells using RNA-Bee reagent according to the manufacturer’s instruction (AMS Bio, Abingdon, UK) and quantified using SpectraMax QuickDrop (Molecular Devices, San Jose, CA, USA). RNA (1 μg) was reverse-transcribed for 50 min at 37 °C in the mixture containing 1 μL oligo (dT), 10 mM dNTP, 0.1 M DTT, 5X PCR buffer and 1μL M-MLV RT (Invitrogen Co., Carlsbad, CA, USA). An aliquot (200 ng) of RT products was amplified in a 25 μL reaction by a GoTaq^®^ Green Master Mix (Promega Co., Madison, WI, USA) in the present of 10 pM oligonucleotide primer. The primers used for RT products from the cells was shown in Table S1. The PCR was performed for 30 cycles at 95 °C for 30 s, 56 °C for 40 s, and 72 °C for 40 s. After amplification, PCR products were separated on 2% agarose gel containing ethidium bromide (Et-Br) by electrophoresis, and the bands were visualized by ultraviolet fluorescence. The experiment was performed in triplicate. The intensities of the bands were analyzed using NIH Image J 1.4 package and normalized to GAPDH value.

### 4.6. Western Blotting

GC-2spd cells were plated at 2 × 10^5^ cells/well and incubated for 24 h. After incubation, the cells were cultured in the presence of various concentrations of apigenin for 2 h, and then stimulated by H_2_O_2_ for 1 h (Sigma-Aldrich Inc., St. Louis, MO, USA). Cells were washed with ice-cold PBS and re-suspended in an appropriate volume of RIPA buffer (50 mM Tris–HCl, pH 7.4, 150 mM NaCl, 1 mM EDTA, 1% NP 40, 0.25% deoxycholate), and incubated for 20 min on ice. Lysates were then centrifuged at 14,000 rpm for 25 min at 4 °C and collected for further analysis. The supernatant was stored at −80 °C until use. Protein concentrations of samples were determined by Bradford assay (Bio-Rad, Hemel, Hempstead, UK) [51]. In each sample, 20 μg of protein was separated on a 10% SDS polyacrylamide gel and transferred to nitrocellulose membrane. Each membrane was incubated for 1 h with 5% skim milk in TBS-T buffer [0.1 M Tris-HCl, pH 7.4, 0.9% NaCl and 0.1% Tween-20] to block non-specific binding, and was then incubated with primary antibodies that recognized GSTm5, GPX4, PRX3, P-CREB, nectin-2 and β-actin (1:1000; Santa Cruz Biotech, Santa Cruz, CA, USA). Each protein was detected by using chemiluminescence detection system according to the instruction of the manufacturer (ECL, Amersham, Berkshire, UK). The experiment was performed in triplicate. The intensities of bands were analyzed by using NIH Image J 1.4 package and normalized to β-actin value.

### 4.7. Statistical Analysis

The results are expressed as the mean ± standard deviation (SD) performed in triplicate. Statistical significance was analyzed by one-way analysis of variance (ANOVA) followed by Tukey’s post hoc test for multiple comparisons using the Graph-Pad prism software package (version 6.0; GraphPad, Inc., La Jolla, CA, USA) for Windows. A value of *p <* 0.05 was considered statistically significant.

## 5. Conclusions

Apigenin isolated from *C. crispus* showed protective effects against testicular cell insufficiency. Apigenin inhibited the level of intracellular ROS in sperm cells and reversed the decreased protein and mRNA expression levels of antioxidant enzymes such as GSTm5, GPX4, and PRX3 in GC-2spd cells exposed to H_2_O_2_. Moreover, apigenin recovered the H_2_O_2_-induced changes in the spermatogenesis associated CREB and nectin-2 molecules in GC-2spd cells. Based on the overall results, apigenin from *C. crispus* might provide an effective and successful strategy in the development of therapeutics aiming at male testicular dysfunctions.

## Data Availability

Data are contained within the article.

## Supplementary Materials

The following are available online at https://www.mdpi.com/article/10.3390/molecules27061777/s1, Figure S1: (a) Bioassay-guided procedure for the isolation of apigenin from *Carduus crispus*, (b) HPLC peak purity, and (c) TLC finger print. Figure S2: (a) 1H-NMR and (b) 13C-NMR spectrum of apigenin. Figure S3: FT-IR spectrum of apigenin. Spectral range: 4000 to 400 cm^−1^, Beam splitter: Ge-coated on KBr, Detector: DTGS, resolution: 0.25 cm^−1^. Figure S4: Mass spectrometry of apigenin. (a) Positive mode, (b) negative mode. Figure S5: UV Spectrum for apigenin. Figure S6: HPLC linearity of apigenin. Table S1: Primers used in the study. Table S2: ^1^H-NMR and ^13^C-NMR data. Table S3: FT-IR spectrum data for apigenin. Table S4: Optical rotation of apigenin. Figure S5: HPLC linearity of apigenin. Table S6: Precision of HPLC 100% method of apigenin.

## Sample Availability

Samples of the compounds are not available from the authors.
