# Supplementary material for: Apigenin Isolated from Carduus crispus Protects against H2O2-Induced Oxidative Damage and Spermatogenic Expression Changes in GC-2spd Sperm Cells
Source: Molecules. 2022 Mar 8;27(6):1777. doi: 10.3390/molecules27061777 (PMC8955133; doi:10.3390/molecules27061777)
Supplement: Supplementary file 1 [file molecules-27-01777-s001.zip › molecules-1559189-supplementary.pdf]

Supplementary files

Apigenin Isolated from *Carduus crispus* Protects against H_2O_2-Induced Oxidative Damage and Spermatogenic Expression Changes in GC-2spd Sperm Cells

Spandana Rajendra Kopalli [1], Sung-Kwang Yoo [2], Bokyung Kim [3], Si-Kwan Kim [4] and Sushruta Koppula [4,*]

1 Department of Bioscience and Biotechnology, Sejong University, Gwangjin-gu, Seoul 05006, Korea; spandanak@sejong.ac.kr
2 Ottugi Food Co., Ltd., Anyang-si 14060, Gyeonggi-do, Korea; kwangbandco@naver.com
3 Department of Physiology and Immunology, School of Medicine, Konkuk University, Chungju 27381, Korea; bkkim2@kku.ac.kr
4 Department of Integrated Biosciences, College of Biomedical & Health Science, Konkuk University, Chungju 27381, Korea; skkim@kku.ac.kr
* Correspondence: sushrutak@gmail.com

1. Materials and Methods

1.1. Identification of Apigenin

Apigenin was identified by the interpretation of 1H- (500 MHz, JEOL) and 13C-NMR (125 MHz JEOL, Tokyo, Japan), FT-IR (Ge-coated KBr pellet, Mattson, Galaxy 7020A), LC/MS (LCQ DECA XP, Thermo Finnigan, Waltham, USA) and UV (Hewlett Packard, photodiode array detector, 269 nm) spectra.

1.2. Purity of Apigenin

Purity of the isolated active ingredient was determined by elemental analysis (Flash EA 1112 series/CE Instrument), Thin-layer chromatographic (TLC) fingerprint and 100% HPLC methods were recorded as further tests for organic contamination.

1.3. Content of Apigenin.

The evaluate the content of apigenin, the plant material of *Carduus crispus* (flower bud) was extracted with 100% methanol for 4 h with reflux system three times. The MeOH extract was evaporated under the reduced pressure and partitioned between EtOAc and water (three times). The EtOAc fractions were pooled, dried *in vacuo* and content of apigenin was analyzed by HPLC (ODS; 250 × 4.6 mm, 5 μm).

1.4. Reverse transcription-polymerase chain reaction (RT-PCR).Primers used in the study was shown in Table S1.

Table S1. Primers used in the study.

Glutathione S-transferase (GST) m5	Forward: 5′-TAT GCT CCT GGA GTT TAC TGA TAC C-3′<br>Reverse: 5′-AGA CGT CAT AAG TGA GAA AAT CCA C-3′
Glutathione peroxidase (GPX) 4	Forward: 5′-GCA AAA CCG ACG TAA ACT ACA CT-3′<br>Reverse: 5′-CGT TCT TAT CAA TGA GAA ACT TGG T-3′
Peroxiredoxin (PRX) 3	Forward: 5′- ACT TTA AGG GAA AAT ACT TGG TGC T-3′<br>Reverse: 5′- TCT CAA AGT ACT CTT TGG AAG CTG T-3′
Nectin-2	Forward: 5′- CAC TAT CAT CAG CCG ATA CTC CT-3′<br>Reverse: 5′- GCT GTA CAG ATG AAG GTA GTG TTG A-3′
CREB	Forward: 5′-ACT GGC TTG GCA CAA CCA GA-3′<br>Reverse: 5′- GGC AGA AGT CTC TTC ATG ATT-3′
Glyceraldehyde-3-phosphate dehydrogenase (GAPDH)	Forward: 5′-AAC TTT GGC ATT GTG GAA GGG C-3′<br>Reverse: 5′-ACA CAT TGG GGG TAG GAA CAC G-3′

2. Results

2.1. Isolation and Identification of Apigenin from Carduus crispus

The isolation procedure was shown in Figure S1a. The peak purity was determined by means of spectral analysis (Figure S1b). Apigenin Rf value in TLC fingerprint was at 0.38, analysed by silica gel TLC plate with the solvent system of hexane:EtOAc:formic acid = 50: 40:5 (Figure S1c).

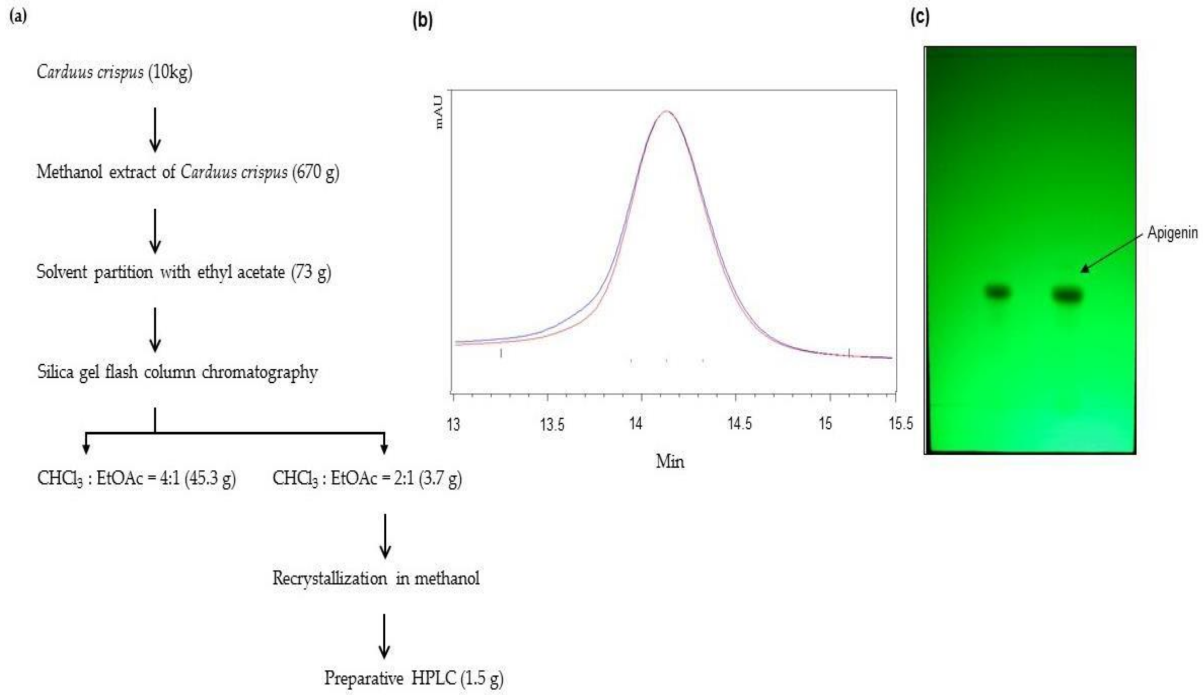

Figure S1. (**a**) Procedure for the isolation of apigenin from *Carduus crispus.* (**b**) HPLC Peak purity and (**c**) TLCfingerprint.

NMR spectra of apigenin was measured as shown in Table S2, respectively and in Figure S2. 1H-NMR (500 MHz, DMSO-d6) spectra (a) and 13C-NMR (125 MHz, DMSO-d6) spectra (b).

^{1}H-NMR (500 MHz, DMSO-*d*$_6$	^{13}C-NMR (125 MHz, DMSO-*d*$_6$
12.95 (1H, s, 5-OH)	181.714 (C-4)
10.80 (1H, s, 7-OH)	163.722 (C-7)
10.39 (1H, s, 4′-OH)	161.427 (C-9)
7.91 (2H, d, j=8.7, H-2′, 6′)	161.153 (C′-4)
6.91 (2H, d, j=8.7, H-3′, 5′)	157.301 (C-5)
6.77 (1H, s, H=3)	128.452 (C2′-6′)
6.48 (1H, s, H=8)	121.158 (C′)
6.18 (1H, s, H6)	115.947 (C3′-5′)
	103.650 (C-10)
	102.819 (C-3)
	98.827 (C-6)
	93.956 (C-8)

Table S2. ^{1}H-NMR and ^{13}C-NMR data (s singlet, d doublet).

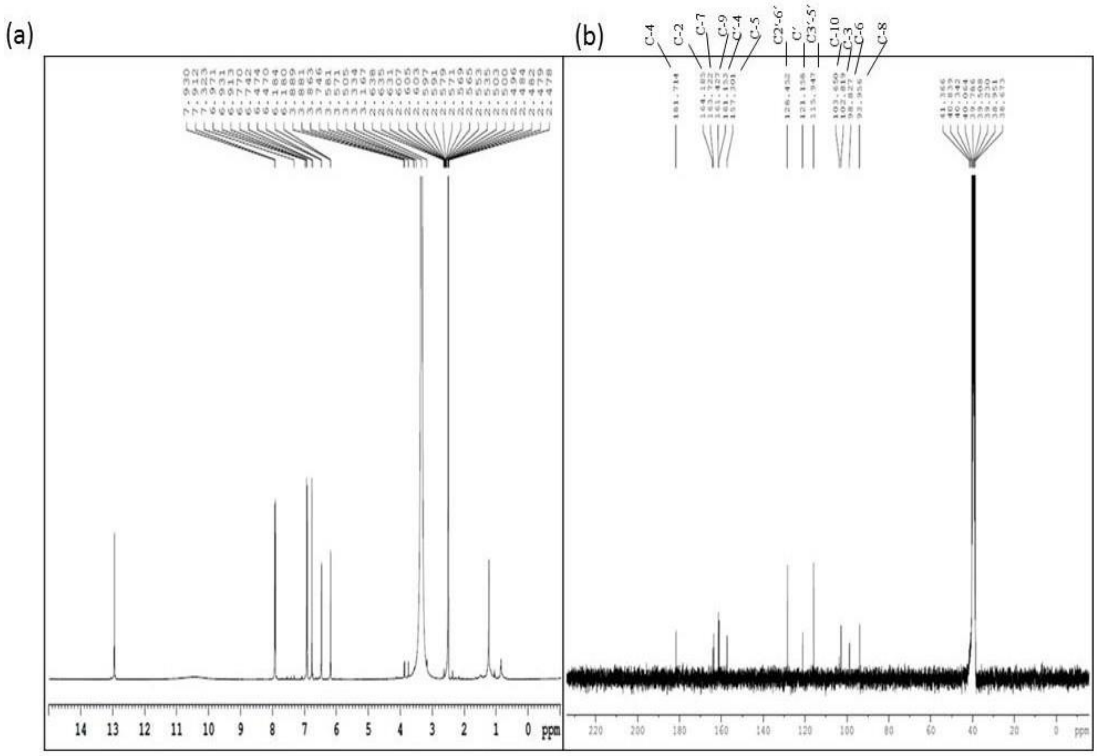

Figure S2. 1H-NMR (**a**) and 13C-NMR (**b**) spectrum of apigenin.

FT-IR of apigenin was measured as shown (Figure S3): Ge-coated KBr pellet (3.0 mgg^{-1}) was measured using a Mattson IR spectroscopy, model Galaxy 7020A. In specific terms, the following correlations could be made as shown in Table S3. Other bands are not significant to the structure.

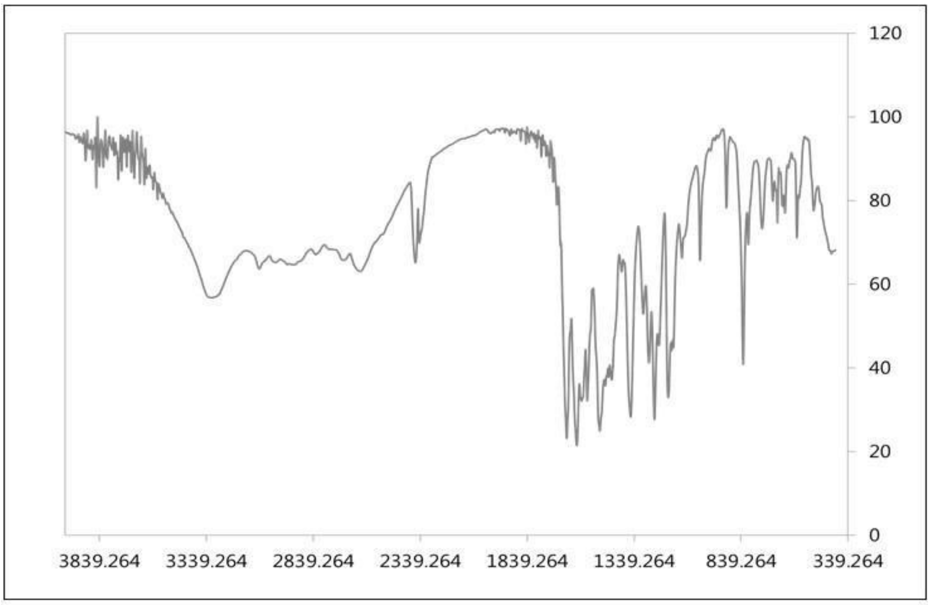

Figure S3. FT-IR spectrum of apigenin. Spectral range: 4000 to 400 cm^{-1}, Beam splitter: Ge-coated on KBr, Detector:DTGS, resolution: 0.25 cm^{-1}.

Chemical moiety	Sample (Wave number: cm^{-1})
γ (O-H): O-H Stretching vibration	3291
γ (C-OH): C-OH Stretching vibration	1354
γ (C=C): C=C Stretching vibration	1608
γ (C=O): C=O Stretching vibration	1651

Table S3. FT-IR spectrum data for apigenin. γ: stretching vibration. FT-IR spectrometer: Mattson Instruments, Inc., Galaxy 7020A, Spectral range: 4000 to 400cm^{-1}, Beam splitter: Ge coated on KBr, detector: DTGS, resolution: 0.25 cm^{-1} (step selectable).

Molar mass of active ingredient was determined to be 269 $[M + H]^-$ in negative mode of LC-MS. But molecular ion peak of active ingredient was not detected in positive mode (Figure S4a and S4b). The UV spectrum of active ingredient took place within the framework of the HPLC analyses with a photodiode array detector. Active ingredient showed with absorption maximum at the vicinity of 207 nm. Also active ingredient showed absorption at 268.5 nm and 336.2 nm (Figure S5).

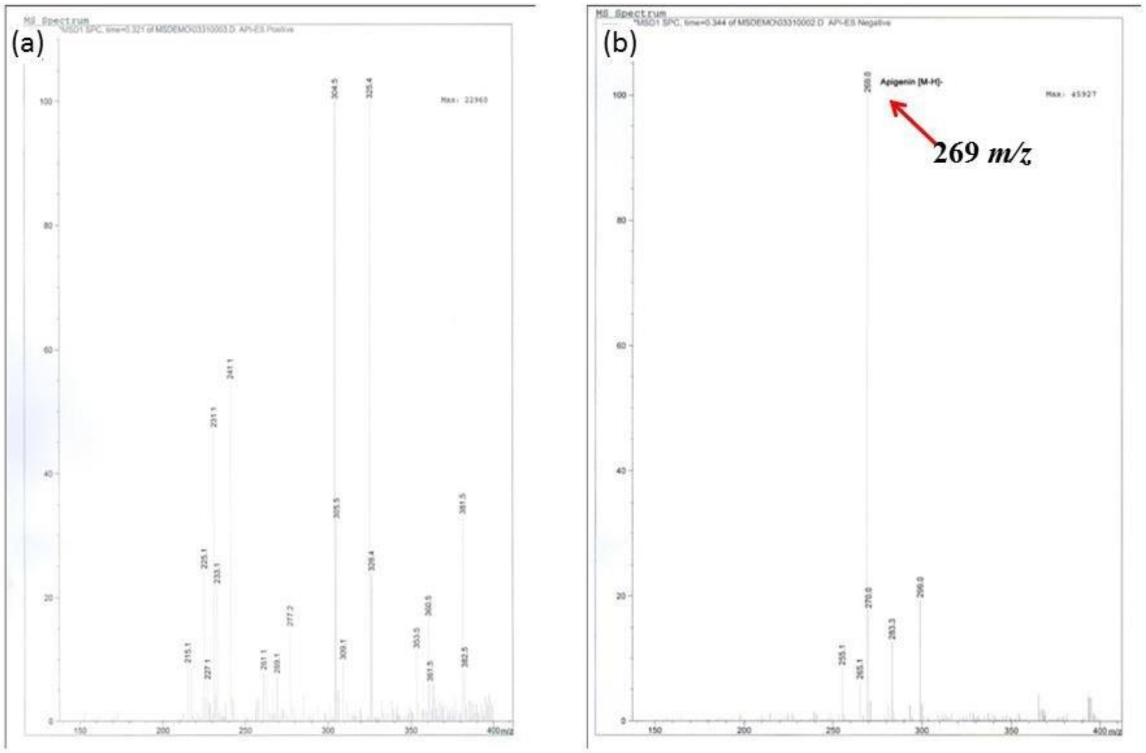

Figure S4. Mass spectrometry of apigenin ingredient. (**a**) positive mode, (**b**) negative mode**.**

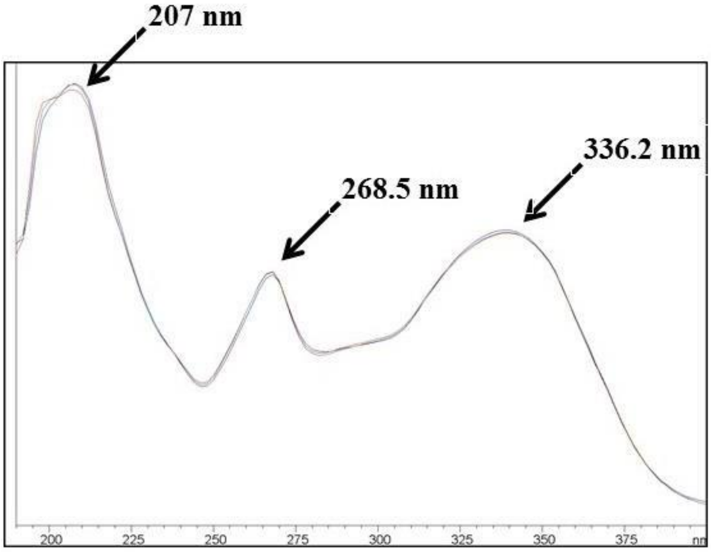

Figure S5. UV Spectrum for apigenin.

Elemental analyses were found to be: carbon 65.36%, hydrogen 3.58%, and nitrogen 0.04%. Optical rotation for active ingredient was analyzed in DMSO, and five duplicates were employed for determination. Isolation of active ingredient was demonstrated $[\alpha]D$ −4.35 in DMSO (Table S4).

Sample No.	Apigenin
1	−4.38
2	−4.33
3	−4.37
4	−4.35
5	−4.32
**Average**	**−4.35**

Table S4. Optical rotation of apigenin. Apigenin in DMSO, Analytical Equipment: JASCO P-1020Polarimetry (Tokyo, Japan), Filter 589 mm, Cylindrical glass cell: 10 × 100 mm.

The linearity of the measuring range for the test substances was verified within the range between 0.0625 mg/ml and 1.000 mg/ml. The regression line is described by the following equation Y = 7309.623X + 39.198 in which Y represents peak areal. Correlation coefficient was R2 = 0.9996 in Figure S6 and Table S5.

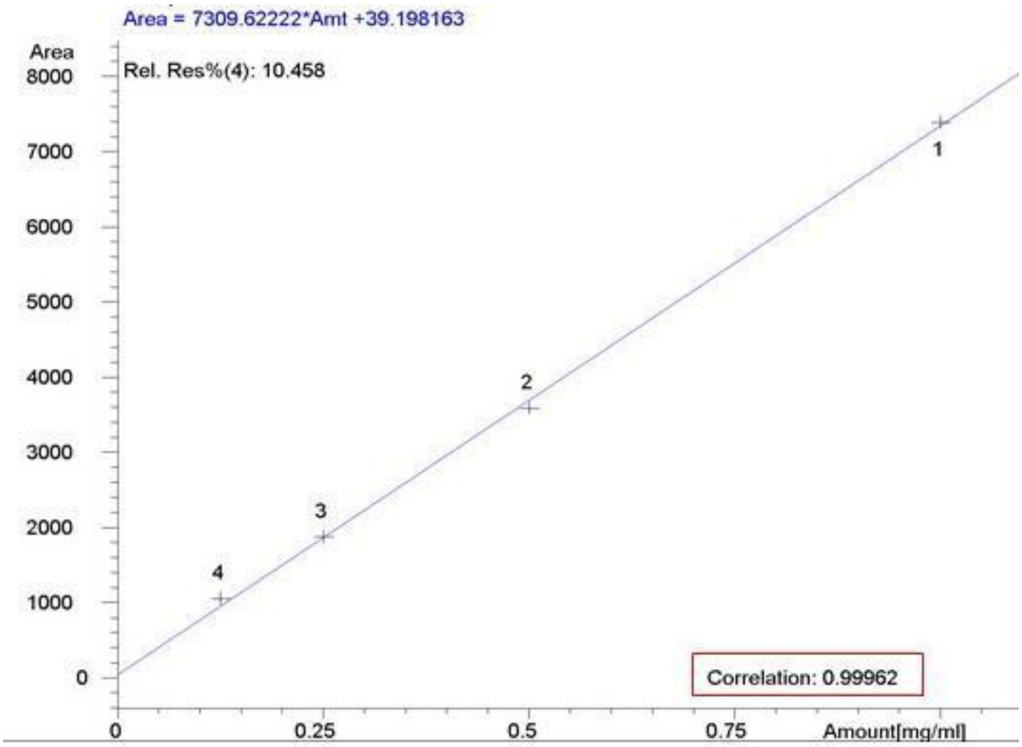

Figure S6. HPLC linearity of apigenin.

Concentration (Apigenin (mg/mL)	Peak areas
0.0625	N/D
0.125	1052.56
0.25	1875.569
0.5	3584.556
1	7388.849

Table S5. HPLC linearity for apigenin. Instrument: Agilent HPLC system, Column: YMC Pack ODS (4.6 × 250 mm),Mobile phase: 70% methanol, flow rate: 1ml/min, detection: UV 269 nm, N/D: not detectable.

Repeatability was shown in Table S6. The content value determined within the framework of sextuple quantitation of apigenin was evaluated to determine the precision. Mean peak retention time of apigenin reference material was 14.22 ± 0.097, and content accounted for 100%.

Measurement	Peak areas	Content (%)
1	2948.6	10
2	2972.0	100
3	2966.2	100
4	2937.4	100
5	2926.6	100
6	2923.9	100
**Peak area mean**	**2945.78 ± 20.145**	

Table S6. Precision of HPLC 100% method for apigenin. Instrument: Agilent HPLC system, column: YMC Pack ODS(4.6 × 250 mm), Mobile phase: 70% methanol, flow rate: 1 ml/min, detection: UV 269 nm.
